# Proposed criteria for nevoid basal cell carcinoma syndrome in children assessed using statistical optimization

**DOI:** 10.1038/s41598-021-98752-9

**Published:** 2021-10-05

**Authors:** Nina B. Gold, Ian M. Campbell, Sarah E. Sheppard, Wen-Hann Tan

**Affiliations:** 1 Division of Medical Genetics and Metabolism, Harvard Medical School, Massachusetts General Hospital for Children, 55 Fruit Street, Boston, MA 02114 USA; 2 Division of Human Genetics, Children’s Hospital of Philadelphia, Philadelphia, PA USA; 3 Department of Biomedical and Health Informatics, Children’s Hospital of Philadelphia, Philadelphia, PA USA; 4 Division of Genetics and Genomics, Boston Children’s Hospital, Harvard Medical School, Boston, MA USA

**Keywords:** Medical genetics, Signs and symptoms

## Abstract

Nevoid basal cell carcinoma syndrome (NBCCS) is a tumor predisposition condition, the cardinal features of which emerge in adolescence or adulthood. Using statistical optimization, this study proposes NBCCS criteria with improved sensitivity in children less than 18 years of age. Earlier detection may lead to improved surveillance and prevention of sequelae. A survey eliciting medical history was completed by, or on behalf of, individuals with NBCCS. Based on these findings, criteria for suspicion of NBCCS in children were suggested using information from a Bernoulli naïve Bayes classifier relying on the human phenotype ontology. The sensitivity and specificity of the existing and proposed diagnostic criteria were also assessed. Participants (n = 48) reported their first signs of NBCCS appeared at a median age of 8 months, but by our retrospective analysis, they did not fulfill the current diagnostic criteria until a median age of 7 years. This study delineates the early-onset features of NBCCS and proposes criteria that should prompt consideration of NBCCS. Additionally, we demonstrate a method for quantitatively assessing the utility of diagnostic criteria for genetic disorders.

## Introduction

Nevoid basal cell carcinoma syndrome (NBCCS, OMIM# 109400), also known as basal cell nevus syndrome, Gorlin syndrome, and Gorlin-Goltz syndrome in some publications, is an autosomal dominant tumor predisposition syndrome associated with a range of congenital anomalies and medical complications. The most recent estimated birth incidence of NBCCS is approximately 1 in 19,000, with a prevalence in the general population of approximately 1 in 31,000. Only 50% of those studied had an identifiable pathogenic variant, however, therefore it is possible that the true incidence and prevalence are lower than these reported rates^1^.

Affected individuals are at risk for malignant and benign tumors, most commonly medulloblastoma and jaw keratocysts during childhood and adolescence, and widespread basal cell carcinomas (BCC) in adulthood^2^. NBCCS is caused by heterozygous loss-of-function pathogenic variants in one of two tumor suppressor genes: *PTCH1* or *SUFU*^3–5^. Because of the varied features associated with NBCCS, clinical criteria are needed to prompt molecular testing. Additionally, clinical criteria are relevant to identifying a possible diagnosis in the approximately 15–27% of affected individuals who meet established diagnostic criteria for NBCCS but do not have a pathogenic variant in either of these associated genes^6,7^. Earlier diagnosis of NBCCS allows for earlier initiation of tumor surveillance, reduction in risk for BCC formation through appropriate protection against exposure to ionizing and UV radiation, and hence improvement in the quality of life^8–10^.

Diagnostic criteria for many genetic disorders have been determined qualitatively, by expert consensus or by designating characteristics observed in case series as diagnostic features^11–14^. A prior consensus statement suggested that a diagnosis of NBCCS should be suspected in the presence of a combination of clinical features, many of which do not emerge until adulthood^2^. Of note, two additional sets of diagnostic criteria have been published previously^15,16^. No prior studies have quantitatively evaluated the utility of the diagnostic criteria for NBCCS, nor have there been proposed criteria for children under 18 years of age.

To improve diagnosis of NBCCS in the pediatric population, we first evaluated the prevalence and temporality of the childhood manifestations and congenital anomalies of NBCCS using an online questionnaire sent to individuals in a NBCCS support group. These data allowed for definition of the natural history, dysmorphic features, and developmental milestones of children with NBCCS. Next, we demonstrated that the existing diagnostic criteria for NBCCS lacked diagnostic sensitivity in childhood. Using our data, we then proposed a set of diagnostic criteria using information from a naïve Bayes classifier (NBC) for NBCCS in children under 18 years of age that had higher sensitivity in our cohort for this age group. To assess the performance of both sets of diagnostic criteria in NBCCS compared with other genetic syndromes, we computationally modeled the specificity of both the existing and proposed diagnostic criteria.

A NBC is a supervised learning algorithm based on Bayes theorem in which the features used to predict the class are considered conditionally independent^17,18^. NBCs are computationally efficient and work well for rare disease because they require relatively small training datasets^17,19^. This methodology may be generalized to generate and assess the sensitivity and specificity of clinical criteria for other genetic conditions that were developed by expert consensus and qualitative methods.

## Results

### Demographic information

A total of 48 participants responded to the online survey (Table 1). Participants included 16 males (33%) and 32 females (67%). The majority of participants self-identified as white (n = 45 [94%]) and non-Latino (n = 40 [83%]). Most participants received the majority of their medical care in the United States (n = 38 [79%]). The median age of participants at time of survey completion was 32 years (range: 5 months to 76 years).

**Table 1:** Demographic characteristics of survey participants (n = 48).

**Sex, N (%)**	
Male	16 (33)
Female	32 (67)
Age in years, median (range)	32 (0–76)
**Race, N (%)**	
White	45 (94)
Black	0 (0)
Asian	1 (2)
American Indian	0 (0)
Pacific Islander	0 (0)
Other	1 (2)
Did not report	1 (2)
**Ethnicity, N (%)**	
Hispanic	7 (15)
Non-Hispanic	40 (83)
Unknown	1 (2)
**Country where medical care was obtained, N (%)**	
United States	38 (79)
Canada	3 (6)
Australia	2 (4)
Colombia	1 (2)
England	1 (2)
New Zealand	1 (2)
Philippines	1 (2)
Spain	1 (2)
**Molecular diagnosis, N (%)**	
*PTCH1*	17 (35)
*SUFU*	2 (4)
Genetic test not completed	19 (40)
Affected family members	12 (25)
No affected family members	6 (13)
Unknown	1 (2)
Genetic test completed, gene unknown	7 (15)
Did not report	3 (6)
**Affected relatives, N (%)**	
1–5 living/deceased affected relatives	18 (38)
6–10 living/deceased affected relatives	3 (6)
> 10 living/deceased affected relatives	3 (6)
No affected relatives	19 (40)
Did not report	5 (10)

Among individuals who reported that they had genetic testing for NBCCS (n = 26, 54%), the majority had a pathogenic variant in *PTCH1* (n = 17, 35%). Two individuals (4%) had a pathogenic variant in *SUFU*. Very few participants reported the specific genetic variants found in themselves or their offspring. Seven individuals (15%) did not know the results of their genetic testing. Among the 19 (40%) individuals who reported that they did not have genetic testing, the majority had clinically affected family members (n = 12). Three individuals did not report whether they had undergone genetic testing. Overall, 24 individuals (50%) reported at least one affected family member, with 3 individuals (6%) reported having more than 10 affected relatives.

### Clinical features of NBCCS

**Figure 1:**
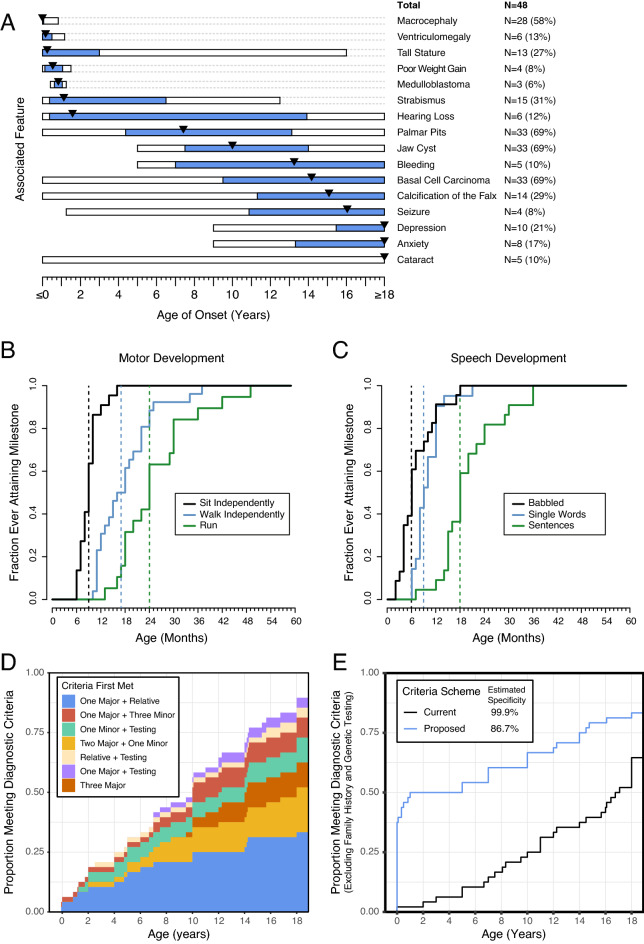
(**A**) Temporal onset of various features of NBCCS. The bars indicate the range of age of onset of each feature, with the blue bar indicating the range of the 25th and 75th percentiles. The black triangle indicates the median age of onset for each feature. Features that occurred prenatally or after age 18 were grouped at ages 0 and 18 respectively. (**B**) Ages at which participants with NBCCS met motor milestones. The black line shows the ages at which patients reportedly met the milestone of sitting independently. The blue line indicates age of walking independently and the green line indicates age of running. The vertical dashed lines of the respective colors indicate the median age of meeting these same milestones. (**C**) Ages at which participants with NBCCS met speech milestones. The black line shows the ages at which patients reportedly met the milestone of babbling. The blue line indicates age of speaking single words and the green line indicates age of speaking full sentences. Not all patients reported data for each milestone resulting in seeming inconsistency in babbling and speaking single words. The vertical dashed lines of the respective colors indicate the median age of meeting these same milestones. (**D**) Ages at which individuals met current diagnostic criteria for NBCCS. The shaded areas indicate the age at which each individual met current diagnostic criteria while the color indicates which combination of citeria the individual met at the earliest age. (**E**) Sensitivity of the current diagnostic criteria as well as proposed criteria across individual age, ignoring family history or genetic testing. The black line indicate the current diagnostic criteria and the blue line indicates the proposed diagnostic criteria. Estimation of the specificity based on Monte Carlo analsysis of simulated individuals generated from the 500 most similar OMIM disease phenotypes is presented in the legend.

The prevalence and temporal onset of features of NBCCS were evaluated (Fig. 1, Supplement 5). The most common clinical features included jaw cysts (n = 33 [69%], median age = 10 years), basal cell carcinomas (n = 33 [69%], median age = 14.2 years), palmar pits (n = 31 [65%], median age = 7.4 years), and macrocephaly (n = 30 [62%], median age = congenital). The age at which patients reported the clinical features of the existing diagnostic criteria was assessed (Table 2).

**Table 2:** Current diagnostic criteria for NBCCS and participants affected with each feature.

Major criteria	Participants affected, N (%)	Median age of onset
Medulloblastoma	3 (6)	10 months
Calcification of falx cerebri	14 (29)	14 years, 6 months
Jaw keratocysts < 20 years old	33 (69)	10 years, 0 months
Palmar or plantar pitting	33 (69)	7 years, 5 months
BCC prior to 20 years old *or* excessive number BCC	33 (69)	14 years, 2 months
First degree relative	12 (25) (participants reported number of total affected relatives; unknown if relatives were first-degree)	Not applicable
**Minor criteria**		
Macrocephaly	28 (58)	1 month
Ocular abnormalities	10 (21)	Neonatal period (presumed)
Cleft lip/palate	5 (10)	Neonatal period (presumed)
Rib anomalies (specifically bifid, splayed, or extra ribs)	20 (42)	Neonatal period (presumed)
Skeletal malformations and radiologic changes	22 (46)	Neonatal period (presumed)
Ovarian or cardiac fibroma	0 (0)	Not applicable
Lymphomesenteric cyst	0 (0)	Not applicable

One major criterion and molecular diagnosis, two major criteria, or one major criterion and two minor criteria are needed to make a diagnosis of NBCCS.

### Developmental milestones

We assessed the age at which participants met motor and speech developmental milestones (Fig. 1B,C). Participants sat independently at a median age of 9 months, walked independently at a median age of 15 months, and ran at a median age of 23 months. Participants babbled at a median age of 6 months, spoke their first words at a median age of 9 months, and spoke in phrases by a median of 19 months.

### Facial dysmorphology

We observed several common facial characteristics that might increase clinical suspicion for NBCCS in children in photographs submitted by participants. The common facial findings observed included down-slanting palpebral fissures, periorbital fullness of the lower eyelids, a flat philtrum, and a distinctive nasal phenotype, characterized by thick nasal alae and a broad columella with high insertion superior to the nasal base.

### Assessment of existing diagnostic criteria

There was a difference of nearly 10 years between the median reported age of onset of symptoms (8 months) and the median age at which the participants reported receiving a diagnoses of NBCCS (11.5 years). In total, 43 out of 48 (90%) individuals met the existing diagnostic criteria at a median age of 7 years. However, excluding the presence of an affected first-degree family member and genetic testing, which are not always available to the clinician, especially when evaluating the first suspected case in a family, only 31 of 48 (65%) individuals reported a history that met the diagnostic criteria at the time at which they completed the survey. The existing diagnostic criteria demonstrated improving sensitivity throughout the lifespan (Fig. 1E, black line), but without the aid of family history or molecular testing, the median age at meeting the diagnostic criteria would be 16.8 years.

### Development and assessment of new clinical criteria

Given the relatively low sensitivity of the existing diagnostic criteria for children in the absence of family history and genetic testing, we sought to propose improved criteria for a pediatric population (Table 3). We developed an NBC using the Bernoulli distribution based on our participant questionnaire data and the simulated individuals with genetic disease. We used the probability data from our model to create novel clinical criteria to prompt consideration for testing of NBCCS in a pediatric population. The scoring system was developed to target a combination of the features seen in the pediatric population in our study.

**Table 3:** Proposed criteria to prompt molecular testing of *PTCH1* and *SUFU*.

Cardinal features (3 points)	Suggestive features (2 points)	Possible features (1 point)
Odontogenic jaw cysts	Macrocephaly (greater than 98th centile)	Tall stature (greater than 98th centile)
Palmar pits	Skeletal anomaly	Structural brain anomalies
Basal cell carcinoma	Medulloblastoma (onset before the age of 5 years and specifically SHH-associated medulloblastoma)	GU anomaly
Calcification of the falx cerebri		Strabismus
Abnormality of the ribs (specifically bifid, splayed, or extra ribs)		Hearing impairment
Congenital structural eye abnormality		Natal teeth
		Oral cleft

Molecular testing is suggested if an individual has features that sum to a total of at least six points (e.g. 2 cardinal features, 3 suggestive features, or 1 cardinal feature with 1 suggestive feature and 1 possible feature). Of note, features that are listed in parentheses are suggested for clinical use but were not included in analyses of sensitivity and specificity as they do not all align with HPO terms.

The *cardinal* features of our criteria are those with onset before or around seven years of age. The *suggestive* features are characteristics seen in at least 5% of the individuals surveyed. The *possible* features are those that are seen commonly in NBCCS, but are non-specific.

These proposed criteria for molecular testing demonstrated improved sensitivity in children compared with the existing clinical criteria (Fig. 1E, blue line). The improvement in sensitivity is particularly notable during the first decade of life. At one year of life, the sensitivity was 50% (24 of 48 participants). The median age at meeting our proposed criteria, without invoking family history or genetic testing, was 6 years of age. With family history, which was assumed to be known at the time of the child’s birth (to conservatively improve the sensitivity of the existing diagnostic criteria), the median age at which the individuals met these diagnostic criteria was 5 months of age. Sensitivity at 7 years of life was 60%. By adulthood, sensitivity was 83% (40 of 48). When compared with the most objectively similar OMIM conditions, the overall specificity of these proposed criteria was 86.7%.

## Discussion

The aims of this study were to elucidate the pediatric characteristics of NBCCS, assess the specificity of the existing diagnostic criteria through the lifespan, and propose more sensitive diagnostic criteria for children less than 18 years of age. To accomplish this, we ascertained survey data from members of a NBCCS support group. We used the survey data, with assistance from a probability classifier, to design a revised set of clinical diagnostic criteria to trigger molecular testing of *PTCH1* and *SUFU* that is more sensitive, though slightly less specific, than the existing criteria for children with NBCCS. For this disorder, we hold that the higher estimated sensitivity is useful for prompting genetic testing, which may confirm a molecular diagnosis of NBCCS.

As a tumor predisposition syndrome, NBCCS is a model disorder in which to improve pediatric diagnostic rates, as earlier diagnosis provides an opportunity for earlier tumor surveillance and implementation of skin cancer prevention strategies. Surveillance guidelines have previously been published, and begin with surveillance for jaw keratocysts as early as 18 months of age^8^. In particular, a growing awareness of the need for limiting sun exposure to reduce the risk of BCC formation and early tumor surveillance may lead to earlier detection of cardiac fibromas, which can cause life-threatening arrhythmias or cardiac failure, or in some cases medulloblastoma^10^. Avoidance of radiation therapy has been recommended for the treatment of medulloblastomas^8^. Radiotherapy and hedgehog pathway inhibition via vismodegib, a medication that treats and prevents BCC formation, may vastly improve morbidity and mortality of NBCCS in the future^8,10,20–23^.

This study defines the temporal onset of signs, symptoms, and developmental milestones of NBCCS. One limitation of our approach is that individuals may have joined a support group because they have more severe manifestations of the condition. Additionally, although all participants reported histories or molecular diagnoses that met the existing clinical criteria, it remains possible that some individuals may not have truly had a diagnosis of NBCCS, thereby falsely increasing the specificity and decreasing the sensitivity of this analysis. Moreover, we could not verify the presence of each clinical feature, and the ages of onset of the various manifestations are subject to recall bias, and it is unclear what effect this had on the performance of the proposed criteria. Nonetheless, the results of our survey suggest that the majority of individuals with NBCCS in this cohort have clinical manifestations from early childhood, but the existing diagnostic criteria do not capture these early manifestations well and hence, these children are often not diagnosed until they develop other clinical features in late childhood or adulthood.

Our data demonstrated a substantial gap between the first reported signs of NBCCS and the age of diagnosis. The majority of participants (71%) self-reported the onset of their features prior to 1 year of life, with median age of 7 months. Our retrospective analysis showed that the median age that individuals met the diagnostic criteria was 4.5 years earlier than their clinical diagnosis (7 years versus 11.5 years of age). In contrast, prior studies found a median age of diagnosis of 19 years in individuals with *PTCH1* variants, and 36 years in individuals with variants in *SUFU*^6^. Using our proposed criteria, the median age at which the affected individuals met the criteria was 5 months.

The major features in the existing NBCCS criteria occur on average in late childhood or adulthood. Jaw cysts and BCC occurred at median ages of 10 and 14.2 years respectively. Additionally, clinical criteria such as bifid ribs, calcification of the falx cerebri, and palmar pits were often identified in affected individuals retrospectively, after the diagnosis of NBCCS had been established, perhaps because they were not assessed on a routine physical exam or would only have been visible on specific X-rays^24^. Many of the same clinical features are included in the prior criteria and our proposed criteria, illustrating that the approaches of criteria based on expert opinion and criteria based on statistical optimization may be complementary. The aid of statistical optimization allows for criteria to be quantitatively and objectively assessed, and iteratively modified to improve its performance.

While it has been recognized that individuals with NBCCS exhibit dysmorphic facial features, these specific phenotypic characteristics found in children have not been described in detail. Using participant photographs, we have delineated characteristic facial features of NBCCS in children to improve the recognizability of the syndrome. This analysis is limited by the lack of racial diversity in this study, which may be due to the fact that African Americans and individuals of Japanese descent with NBCCS are less likely to develop BCCs^16,25^.

Additionally, we substantiated prior observations that many young children with NBCCS have early motor delays^26^. Other studies have found intellectual disability in up to 17.6% in Japan with NBCSS, which was not replicated in this study^25^. Among individuals with NBCSS of Japanese descent, deletion of the entire *PTCH1* gene is the molecular diagnosis in 16% of cases^27^. Given that the contiguous gene deletion syndrome of 9q22.3 has a similar phenotype to NBCCS, but also includes intellectual disability, it is plausible that whole gene deletion may lead to a more severe phenotype than single nucleotide variants or small deletions and duplications within *PTCH1,* thereby providing a possible explanation for the discrepant findings in the rate of intellectual disability these populations^27,28^. Importantly, because a minority of children with NBCSS may have developmental delay or intellectual disability, they may be less likely to benefit from the new evidence-based guidelines regarding the use of whole exome sequencing as a first-tier diagnostic genetic test, highlighting the importance of clinical criteria to prompt genetic testing^29^.

We undertook a novel approach to the development and evaluation of a new clinical criteria to prompt genetic testing and consideration of NBCCS, which has improved sensitivity for NBCCS in a pediatric population. Beyond NBCCS, our approach is informed by probability models and clinical expertise, then subsequently assessed for sensitivity and specificity. Importantly, the approach we developed to create these criteria will have utility in other disorders. While our approach relied heavily on accurate, temporal phenotypic data about the participants in our study, if similarly detailed data were collected for individuals with other genetic disorders, this method could easily be adapted to construct other clinical and diagnostic criteria schemes.

This approach relied on simulated individuals with genetic disease and is not subject to the bias of clinicians or recall bias of families. However, if detailed, phenotypic data with age of onset could be collected for a broad cohort of individuals with suspected genetic disease, the specificity of proposed criteria could be more accurately assessed against actual participants with a range of genetic conditions. Additionally, some clinical features of NBCCS, such as rib abnormalities may be relatively common in individuals who do not have genetic syndromes, but this analysis does not address how specific the proposed criteria is in the general population, as we are unaware of a database or resource that provides deep phenotyping of healthy individuals using HPO terms, which would be necessary for such analyses^30^. This would be an important area for further research.

In summary, it is imperative to identify the tumor predisposition syndrome NBCCS in the pediatric population to facilitate improved tumor surveillance and management. In turn, these prevention strategies may mitigate the onset of depression and anxiety which were common in our study, and are potentially related to complications of this disease. Our findings suggest that the majority of participants with NBCCS have early childhood-onset clinical features, which are sometimes unrecognized by the existing diagnostic criteria. With the assistance of statistical optimization, we demonstrated that new criteria, which can be used to prompt molecular testing, may improve sensitivity while maintaining clinically useful specificity in comparison to the prior diagnostic criteria. These new pediatric criteria for NBCCS may facilitate early consideration of NBCCS and thus improve tumor prevention and management in affected children. Moreover, this model of statistical optimization used in concert with criteria created by expert consensus may be generalizable to the design of clinical criteria in other genetic conditions.

## Methods

### Data source and participant population

We conducted a retrospective study of individuals with NBCCS through an online questionnaire, which included questions pertaining to participant demographics, genetic testing, developmental milestones, and medical history (Supplement 1).

Participants were recruited through the BCCNS Life Support Network (now known as the Gorlin Syndrome Alliance) via an email and a Facebook post inviting participants to complete the survey^31^. Informed consent was obtained from a parent and/or legal guardian for study participation. Participants included in the analysis provided a virtual signature. All methods were carried out in accordance with relevant guidelines and regulations and data have been de-identified.

A total of 79 participants responded. Participants were excluded if they did not provide an online signature to consent for participation (n = 18) or left all survey responses blank (n = 4). Only individuals who met existing clinical criteria and/or had a molecular diagnosis of NBCCS were included (n = 48). Entries submitted by individuals with the same name and date of birth were consolidated to represent one entry; when an individual provided discrepant answers in two distinct submissions, the answer with the later time stamp was included (n = 18). Some participants reported the presence of a feature but did not provide an age of onset; in these cases, the feature was reported in the total count but was excluded from age-based analysis. Descriptive statistics were used to describe demographics. Median ages were used to describe the onset of clinical features.

### Assessment of sensitivity of existing diagnostic criteria

For the majority of clinical features, participants were asked for the age of diagnosis. For congenital anomalies, the age of onset was assumed to be prenatal or in the neonatal period (both coded as 0 months of life). We applied the existing diagnostic criteria to the data provided by each individual participant and assigned the age at which the individual met these criteria.

Because our questionnaire did not explicitly address when the family members of a participant were diagnosed with NBCCS and how they were related to the participant, it was assumed that affected relatives were first-degree and had been diagnosed prior to the child’s birth. This assumption conservatively improved the sensitivity of the existing diagnostic criteria compared with the proposed diagnostic criteria, as only the existing diagnostic criteria includes affected relatives as a suggestive clinical feature. Sensitivity of the criteria was evaluated at monthly intervals between birth and 18 years of age.

### Development of new clinical criteria

We hypothesized that the proposed diagnostic criteria may have improved sensitivity for children with NBCCS under 7 years of age. We developed a simulated population of individuals with genetic diagnoses against which the ability of the current and the proposed criteria to discriminate between individuals with NBCCS and other individuals referred for clinical genetics evaluation was assessed. Fifty thousand simulated control individuals were sampled from OMIM phenotype Human Phenotype Ontology (HPO) collections using the R statistical programing language^32^. The 500 most similar OMIM phenotypes to NBCCS were calculated via Resnik similarity using the Ontology Similarity package^33^. A NBC using the Bernoulli distribution was trained using the fastNaiveBayes package. The effectiveness of the classifier was assessed using tenfold cross validation. Using probability and ratio output from the classifier model, we developed multiple sets of revised diagnostic criteria. These methods are summarized graphically in Supplement 2 (created with Adobe Illustrator version 25.2).

To assess the sensitivity and specificity of these revised criteria, we implemented them in R (Supplement 3). Some phenotypes are considered present or absent and are represented by true/false. Others are accumulated over time and are denoted as an age in months of development.

To implement the current criteria (Table 2), major criteria were coded as HP:0010603, HP:0010610, HP:0005462, and HP:0002671. Minor criteria were coded as HP:0002885; HP:0000256; HP:0000175 or HP:0410030, HP:0010442; HP:0000925 or HP:0000772; and HP:0000518 or HP:0000589 or HP:0000568 or HP:0008058. No individual reported fibromas of the ovaries or heart. A diagnosis was made when a participant exhibited two major criteria and one minor criterion, or one major criterion and three minor criteria^2^.

We implemented our proposed criteria (Table 3) as follows. Cardinal features were coded as HP:0010603; HP:0010610; HP:0005462; HP:0002671; HP:0000772; and HP:0000518 or HP:0000589 or HP:0000568 or HP:0008058. Suggestive features were coded as HP:0002885, HP:0000256, and HP:0000925. Possible features were coded as HP:0000098; HP:0002119 or HP:0002126 or HP:0002308 or HP:0100702; HP:0000486; HP:0000695; HP:0000175 or HP:0410030; HP:0000107 or HP:0000104; and HP:0000365. Points were assigned for each feature, 3 points per cardinal feature, 2 points per suggestive feature, and 1 point per possible feature. Molecular testing of *PTCH1* and *SUFU* is suggested when a sum of 6 or more points are tabulated. Separately, molecular testing is also suggested when there is an affected first-degree relative. Since we consider an affected first-degree relative to be sufficient to prompt testing, this was not included as a feature in the proposed criteria.

The sensitivity of each set of proposed criteria was calculated at each age.

In an attempt to assess the specificity of the current criteria, we developed a control set of simulated individuals using phenotype information annotated to diseases in OMIM (Supplement 4). We generated 50,000 such simulated individuals by sampling a random number of features among all annotated features. A feature was marked as “present” if the HPO term or any of its descendants in the HPO hierarchy was present in the simulated individual's list of HPO terms. To more closely mirror the clinical diagnostic process, we limited our control set to the 500 diseases most similar to NBCCS based on Resnik similarity, a score denoting the frequency of shared HPO terms. Because OMIM data do not provide sufficient data on the age of onset of phenotypes, we only assessed specificity at adulthood. When compared to the 500 disorders with the highest Resnik similarity, the overall specificity was calculated by dividing the number of simulated individuals who did not meet criteria for NBCCS (true negatives) by the same number plus the simulated individuals with other genetic disorders who did meet NBCCS criteria (false positives).

### Ethics approval

This study was approved by the Institutional Review Board of Boston Children’s Hospital.

### Consent to participate

Informed consent was required from participants (if over 18 years of age), or from a parent or legal guardian (if below 18 years of age). Participants included in the analysis provided a virtual signature. Data have been de-identified.

## Supplementary Information

Supplementary Information 1.

Supplementary Information 2.

Supplementary Information 3.

Supplementary Information 4.

Supplementary Information 5.

Supplementary Information 6.

## Data Availability

Our anonymized clinical data is available for sharing.

## Code availability

The code, implemented in R, used to assess the sensitivity and specificity of the diagnostic criteria are available in Supplement 3.
