# Supplementary material for: Proposed criteria for nevoid basal cell carcinoma syndrome in children assessed using statistical optimization
Source: Sci Rep. 2021 Oct 5;11:19791. doi: 10.1038/s41598-021-98752-9 (PMC8492651; doi:10.1038/s41598-021-98752-9)
Supplement: Supplementary file 1 — Supplementary Information 1. [file 41598_2021_98752_MOESM1_ESM.docx]

**Supplemental materials**

Supplement 1. Questionairre provided to participants.

##### Supplement 2. We surveyed the Gorlin Syndrome Support group and received 48 responses (left column of figure, see Supplement 1 for actual survey and Supplement 3 for the results). We standardized the results of our online survey using Human Phenotype Ontology (HPO) terms for each participant (blue table). We also recorded the age at which they each participant first reported the sign or symptom first.

To create a control set of patients, we extracted the HPO terms annotated to all of the phenotypes of genetic syndromes listed in OMIM (pink table, right column of figure). Note that there is no age of onset information available for these annotations.

### Next, we used HPO frequency information from our survey and frequency information and information content from the OMIM annotations to create a Bernoulli naïve Bayes classifier (center column). We used information from the probability distributions of this classifier along with the survey results to inform our design of the proposed diagnostic criteria. We then used the survey data to generate age-specific sensitivity estimates for our criteria.

To create an estimate of specificity we used Resnik similarity and the HPO annotations to generate a list of the 500 genetic syndromes with the highest phenotypic similarity to basal cell carcinoma nevus syndrome. We selected the 500 phenotypes most similar to BCCN to more closely mimic the process of differential diagnosis (orange table). Using random sampling, we then created 50,000 simulated patients, each with an assortment of phenotypes representative of an underlying OMIM disease (green table). We used these simulated patients to estimate the specificity of our proposed diagnostic criteria.

Supplement 3. Results of survey.

Supplement 4. 500 most similar Online Mendelian Inheritance in Man (OMIM) phenotypes, ranked by Resnik similarity.

Supplement 5. Ages of onset of characteristics in individuals with nevoid basal cell carcinoma syndrome.
