# Supplementary material for: Proposed criteria for nevoid basal cell carcinoma syndrome in children assessed using statistical optimization
Source: Sci Rep. 2021 Oct 5;11:19791. doi: 10.1038/s41598-021-98752-9 (PMC8492651; doi:10.1038/s41598-021-98752-9)
Supplement: Supplementary file 2 — Supplementary Information 2. [file 41598_2021_98752_MOESM2_ESM.pdf]

Resize font:

|

Introduction

Page 2 of 6

DEMOGRAPHICS

Throughout the survey, the "participant" refers to the person with BCCNS. If you are completing this survey on behalf of your child or another family member, please answer the following questions as they refer to that individual.

Participant's date of birth (MM-DD-YYYY) Today M-D-Y

Participant's sex
- ◯ Male
- ◯ Female

reset

Participant's ethnicity
- ◯ Hispanic or Latino
- ◯ Not Hispanic or Latino
- ◯ Unknown

reset

Participant's race *(Check all that apply.)*
- ☐ White
- ☐ Black / African-American
- ☐ Asian
- ☐ Native American / Alaskan Native
- ☐ Native Hawaiian / Other Pacific Islander
- ☐ Other

In what country has the participant received most of his/her medical care?

<< Previous Page

Next Page >>

Save & Return Later

Introduction

Resize font:

Page 5 of 6

DEVELOPMENT

Has the participant ever required Early Intervention services?

○ Yes
○ No

reset

If the participant is >3 years of age, have they ever required special education classes or an individualized learning plan (IEP)?

○ Yes
○ No

reset

Does the participant have any other special learning needs?

○ Yes
○ No

reset

Has the participant ever been diagnosed with an autism spectrum disorder?

○ Yes
○ No

reset

Please indicate the age at which the participant reached these developmental milestones.

LANGUAGE

Babbled (made sounds like "gaga" or "baba")

Spoke first word

Spoke in sentences with 2 to 4 words

Told a simple story

MOTOR

Rolled in both directions (front to back and back to front)

Sat unassisted

Crawled

Walked

Ran

<< Previous Page

Next Page >>

Save & Return Later

Resize font:

Introduction

Page 3 of 6

GENETICS

Has the participant had genetic testing for BCCNS?

- Yes
- No

reset

Does/did the participant have any biological relatives with BCCNS?

- Yes
- No

reset

If you have a genetic testing report for the participant or one of the participant's family members with BCCNS, please upload it here.

Upload document

At what age (in years and/or months) was the participant diagnosed with BCCNS?

At what age (in years and/or months) do you believe that the participant first showed any signs or symptoms of BCCNS?

<< Previous Page

Next Page >>

Save & Return Later

Resize font:

Introduction

Page 4 of 6

MEDICAL HISTORY

Before the participant was born, were any abnormalities found on fetal ultrasound or other prenatal imaging (i.e., MRI or echocardiogram)?

- ○ Yes
- ○ No

reset

Was the participant born full-term or premature?

- ○ Full-term
- ○ Premature

reset

Did the participant have any of these findings related to pregnancy?

- ☐ Intrauterine growth restriction (small fetus)
- ☐ Macrosomia (large fetus)
- ☐ Oligohydramnios (not enough amniotic fluid)
- ☐ Polyhydramnios (too much amniotic fluid)
- ☐ None of these findings
- ☐ Unknown

Has the participant ever had any of these conditions related to growth?

- ☐ Difficulty gaining weight (sometimes called "failure to thrive")
- ☐ Macrocephaly (large head)
- ☐ Microcephaly (small head)
- ☐ Short stature
- ☐ Tall stature
- ☐ None of these conditions
- ☐ Unknown

What is the participant's current height? Please specify the unit of measurement in your answer.

What is the participant's current weight? Please specify the unit of measurement in your answer.

If known, what is the circumference of the patient's head in centimeters (as measured at a doctor's office)?

Has the participant ever had cancer?

- ☐ Basal cell carcinoma
- ☐ Leukemia
- ☐ Medulloblastoma
- ☐ Melanoma
- ☐ Squamous cell carcinoma
- ☐ Rhabdomyoma
- ☐ Other cancer
- ☐ No, the participant has never had cancer
- ☐ Unknown

Does the participant have any of these conditions related to the skin?

- ☐ Atypical birthmark
- ☐ Palmar pits (small pinpoint-sized holes in the palms)
- ☐ Other skin condition
- ☐ No, the participant has not had any skin conditions
- ☐ Unknown

Does the participant have any of these conditions related to the face and teeth?

- ☐ Cleft lip
- ☐ Cleft palate
- ☐ Extra teeth
- ☐ Jaw cyst
- ☐ Natal tooth (teeth present at birth)
- ☐ Other condition of the face or teeth
- ☐ No, the participant has not had any conditions of the face or teeth
- ☐ Unknown

Has the participant had any of these conditions related to the eyes?

- ☐ Anophthalmia (absent eye)
- ☐ Blindness
- ☐ Cataract
- ☐ Coloboma
- ☐ Glaucoma
- ☐ Microophthalmia (small eye)
- ☐ Myelinated optic nerve
- ☐ Poor vision
- ☐ Strabismus (sometimes called a "lazy eye")
- ☐ Other eye abnormalities
- ☐ No, the participant has not had any eye conditions
- ☐ Unknown

Has the participant had any of these conditions related to the ears?

- ☐ Anotia (absent ear)
- ☐ Hearing loss
- ☐ Microtia (small ear)
- ☐ Other ear abnormalities
- ☐ No, the participant has not had any ear conditions
- ☐ Unknown

Has the participant had any of these conditions related to the brain or spine?

- ☐ Arachnoid cyst (small fluid-filled cyst on brain, typically seen on prenatal ultrasound)
- ☐ Calcification of the falx cerebri
- ☐ Chiari malformation (cerebellum is shifted downward)
- ☐ Dandy-Walker syndrome (absent or small cerebellum)
- ☐ Encephalocele (brain tissue outside the skull)
- ☐ Movement disorder (e.g., dystonia, dyskinesia)
- ☐ Neural tube defect (i.e. spina bifida)
- ☐ Polymicrogyria (abnormal folding pattern of brain)
- ☐ Seizures
- ☐ Ventriculomegaly (large ventricles of the brain)
- ☐ Other brain or spine abnormality
- ☐ No, the participant has not had any conditions of the brain or spine
- ☐ Unknown

Has the participant ever had any of these conditions related to the heart?

- ☐ Aortic valve stenosis
- ☐ Atrial septal defect
- ☐ Cardiac fibroma
- ☐ Cardiomyopathy (problem with heart function)
- ☐ Coarctation of the aorta
- ☐ Complete atrioventricular canal defect
- ☐ Ebstein anomaly
- ☐ Hypoplastic left heart syndrome
- ☐ Pulmonary valve stenosis
- ☐ Tetralogy of Fallot
- ☐ Total anomalous pulmonary venous connection
- ☐ Truncus arteriosus
- ☐ Ventricular septal defect
- ☐ Other heart anomaly
- ☐ No, the participant has never had a heart condition
- ☐ Unknown

Has the participant had any of these conditions related to the lungs?

- ☐ Congenital cystic adenomatoid malformation (also known as CCAM, a lung cyst)
- ☐ Congenital diaphragmatic hernia
- ☐ Other lung condition
- ☐ No, the participant has never had a lung condition
- ☐ Unknown

Has the participant had any of these conditions related to the gastrointestinal system?

- ☐ Duodenal atresia
- ☐ Esophageal atresia or tracheo-esophageal fistula
- ☐ Gastroschisis
- ☐ Imperforate anus
- ☐ Jejunoileal atresia
- ☐ Malformation of the liver
- ☐ Malformation of the pancreas
- ☐ Omphalocele
- ☐ Other type of gastrointestinal condition
- ☐ No, the participant has never had a gastrointestinal condition
- ☐ Unknown

Has the participant ever had any of these conditions related to the genitourinary system?

- ☐ Absent or underdeveloped LEFT kidney
- ☐ Absent or underdeveloped RIGHT kidney
- ☐ Ambiguous genitalia
- ☐ Cloacal exstrophy
- ☐ Cryptorchidism (undescended testicles)
- ☐ Horseshoe kidney
- ☐ Imperforate hymen
- ☐ Kidney cyst
- ☐ Other type of kidney, bladder, or genital problem
- ☐ No, the participant has never had a condition of the kidneys or bladder
- ☐ Unknown

Has the participant ever had any of these conditions of the bones?

- ☐ Abnormalities of ribs (such as "bifid ribs")
- ☐ Abnormalities of vertebrae (abnormally-shaped spinal bones)
- ☐ Polydactyly of feet (extra toes)
- ☐ Polydactyly of hands (extra fingers)
- ☐ Syndactyly of feet (webbed toes)
- ☐ Syndactyly of hands (webbed fingers)
- ☐ Other bone problem
- ☐ No, the participant has not had any bone conditions
- ☐ Unknown

Has the participant ever had any of these conditions of the blood?

- ☐ Abnormal bleeding or bruising
- ☐ Blood clots
- ☐ No, the participant has not had any conditions of the blood
- ☐ Unknown

Has the participant ever had any of these psychiatric conditions?

- ☐ Anxiety
- ☐ Bipolar disorder
- ☐ Depression
- ☐ Obsessive compulsive disorder
- ☐ Psychosis or schizophrenia
- ☐ No, the participant has never been diagnosed with a psychiatric condition
- ☐ Unknown

<< Previous Page

Next Page >>

Save & Return Later

Resize font:

Introduction

Page 6 of 6

PHOTOS

Please upload a facial photograph of the participant.

If the photo is too large to upload, please send it directly to Dr. Wen-Hann Tan at wen-hann.tan@childrens.harvard.edu.

Note: If we identify a basal cell carcinoma or other concerning skin finding in your photograph, we may contact you by email to recommend an evaluation with your local physician.

Upload document

What age is the participant in photo?

<< Previous Page

Submit

Save & Return Later
