# Supplementary material for: Proposed criteria for nevoid basal cell carcinoma syndrome in children assessed using statistical optimization
Source: Sci Rep. 2021 Oct 5;11:19791. doi: 10.1038/s41598-021-98752-9 (PMC8492651; doi:10.1038/s41598-021-98752-9)
Supplement: Supplementary file 3 — Supplementary Information 3. [file 41598_2021_98752_MOESM3_ESM.pdf]

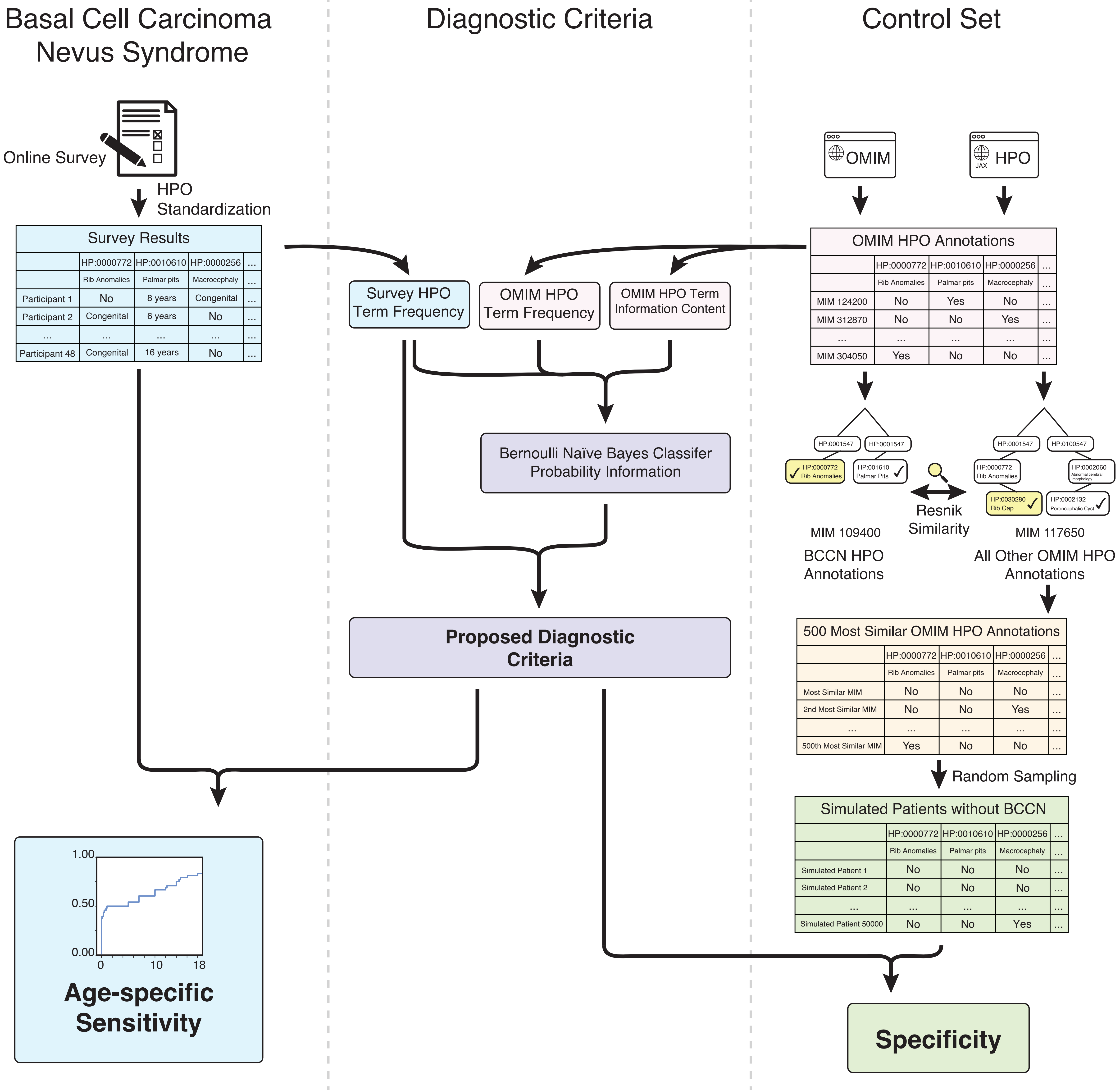
Basal Cell Carcinoma Nevus Syndrome
Diagnostic Criteria
Control Set
Online Survey
HPO Standardization
Survey Results
HP:0000772 HP:0010610 HP:0000256 ...
Rib Anomalies Palmar pits Macrocephaly ...
Participant 1 No 8 years Congenital ...
Participant 2 Congenital 6 years No ...
... ...
Participant 48 Congenital 16 years No ...
OMIM
HPO
JAX
OMIM HPO Annotations
HP:0000772 HP:0010610 HP:0000256 ...
Rib Anomalies Palmar pits Macrocephaly ...
MIM 124200 No Yes No ...
MIM 312870 No No Yes ...
... ...
MIM 304050 Yes No No ...
Survey HPO Term Frequency
OMIM HPO Term Frequency
OMIM HPO Term Information Content
Bernoulli Naïve Bayes Classifer Probability Information
HP:0001547
HP:0001547
HP:0000772 Rib Anomalies
HP:001610 Palmar Pits
HP:0001547
HP:0100547
HP:0000772 Rib Anomalies
HP:0002060
HP:0030280 Rib Gap
HP:0002132 Porencephalic Cyst
Resnik Similarity
MIM 109400
MIM 117650
BCCN HPO Annotations
All Other OMIM HPO Annotations
Proposed Diagnostic Criteria
500 Most Similar OMIM HPO Annotations
HP:0000772 HP:0010610 HP:0000256 ...
Rib Anomalies Palmar pits Macrocephaly ...
Most Similar MIM No No No ...
2nd Most Similar MIM No No Yes ...
... ...
500th Most Similar MIM Yes No No ...
Random Sampling
Simulated Patients without BCCN
HP:0000772 HP:0010610 HP:0000256 ...
Rib Anomalies Palmar pits Macrocephaly ...
Simulated Patient 1 No No No ...
Simulated Patient 2 No No No ...
... ...
Simulated Patient 50000 No No Yes ...
1.00
0.50
0.00
0
10
18
Age-specific Sensitivity
Specificity
