# Supplementary material for: Proposed criteria for nevoid basal cell carcinoma syndrome in children assessed using statistical optimization
Source: Sci Rep. 2021 Oct 5;11:19791. doi: 10.1038/s41598-021-98752-9 (PMC8492651; doi:10.1038/s41598-021-98752-9)
Supplement: Supplementary file 6 — Supplementary Information 6. [file 41598_2021_98752_MOESM6_ESM.docx]

Supplement 5. Ages of onset of characteristics in individuals with nevoid basal cell carcinoma syndrome.

| **Clinical history** | **Affected individuals, N** | **Median age of diagnosis** |
| --- | --- | --- |
| Prenatal finding  Macrosomia  IUGR | 2  1 | Prenatal  Prenatal |
| Growth  Macrocephaly  Tall stature  Poor weight gain  Short stature | 28  13  4  2 | 1 month  1 month  7 months  13 years, 7 months |
| Cancer  Basal cell carcinoma  Squamous cell  carcinoma  Medulloblastoma  Melanoma  Rhabdomyoma  Breast cancer  Non-Hodgkins  lymphoma | 33  5  3  1  1  1  1 | 14 years, 2 months  18 years or older  10 months  18 years or older  7 months  18 years or older  16 years, 0 months |
| Skin  Palmar pits  Psoriasis  Eczema  Actinic keratosis  Atypical birthmark    Folliculitis  Sebaceous cysts  Hemangioma  Histiocytoma | 33  2  2  1  1  1  1  1  1 | 7 years, 5 months  14 years, 6 months  13 years, 0 months  18 years or older  Neonatal period (presumed)  15 years, 1 month  5 years, 0 months  5 years, 0 months  5 years, 0 months |
| Craniofacial  Jaw cyst  Absent teeth  Cleft lip and palate    Cleft lip    Supernumerary teeth  Natal tooth    Hypertelorism  Cleft palate    Macrognathia | 33  3  2  2  2  2  2  1  1 | 10 years, 0 months  2 months  Neonatal period (presumed)  Neonatal period (presumed)  Unknown  Neonatal period (presumed)  Unknown  Neonatal period (presumed)  12 years, 0 months |
| Eye  Strabismus  Low visual acuity  Cataract  Glaucoma  Coloboma    Microophthalmia    Extropia  Myelinated optic nerve  Optic nerve hypoplasia  Nevus of eye  Weak superior rectus  Lacrimal duct  obstruction | 15  9  5  4  2  2  2  1  1  1  1  1 | 1 year, 2 months  Unknown  18 years or older  18 years or older  Neonatal period (presumed)  Neonatal period (presumed)  2 months  Unknown  4 months  18 years or older  6 years, 0 months  4 months |
| Ear  Hearing loss  Low-set ears  Hyperacusis  Small ear canals | 6  1  1  1 | 1 year, 7 months  Unknown  Unknown  18 years or older |
| Neurologic  Calcification of falx  Ventriculomegaly  Seizures  Chiari malformation  Neural tube defect    Hydrocephalus  Arachnoid cyst  Movement disorder  Polymicrogyria  Ventricular cyst  Tarlov cyst  En plaque meningioma  Basilar artery  malformation | 14  6  4  2  2  2  1  1  1  1  1  1  1 | 14 years, 6 months  2 months  16 years, 1 month  Unknown  Neonatal period (presumed)  Prenatal  Prenatal (presumed)  18 years or older  Prenatal (presumed)  6 months  18 years or older  18 years or older  18 years or older |
| Cardiac  Cardiomyopathy  Tricuspid valve  regurgitation  Pericardial cyst  Aortic aneurysm | 2  1  1  1 | 18 years or older  Unknown  Unknown  Unknown |
| Pulmonary  Unilateral absent lung  Bronchogenic cyst  Pneumothorax | 1  1  1 | Prenatal  Prenatal  18 years or older |
| Gastrointestinal  IBS  Liver cysts  Pancreatic cysts  Diverticulosis | 2  1  1  1 | 18 years or older  Neonatal period  Neonatal period  18 years or older |
| Genitourinary  Renal cyst  Uterine fibromas  Absent left kidney  Absent right kidney  Absent kidney (side  unknown)  Cryptorchidism  Bicornuate uterus  Vesicoureteral reflux  Medullary sponge kidney  Imperforate hymen  Ovarian cysts  Endometriosis | 4  2  2  2  1  1  1  1  1  1  1  1 | 18 years or older  18 years or older  Prenatal (presumed)  Prenatal (presumed)  Prenatal (presumed)  Neonatal period  Unknown  Prenatal  17 years, 2 months  14 years, 0 months  18 years or older  18 years or older |
| Musculoskeletal  Rib abnormalities  Scoliosis  Vertebral abnormalities  Syndactyly of toes    Syndactyly of fingers    Polydactyly of hand    Pectus excavatum  Pes planus  Leg length discrepancy  Endochondroma  Osteoporosis | 20  8  4  4  1  1  1  1  1  1  1 | Unknown  6 years, 0 months  Unknown  Neonatal period (presumed)  Neonatal period (presumed)  Neonatal period (presumed)  Unknown  3 years, 0 months  Unknown  18 years or older  18 years or older |
| Hematologic  Abnormal bleeding  Blood clot | 5  1 | 13 years, 3 months  17 years, 3 months |
| Psychiatric  Depression  Anxiety  Bipolar disorder  OCD | 10  8  1  1 | 18 years or older  18 years or older  18 years or older  9 years, 4 months |
